# Regional Differences in the Frequency of *BRCA1* and *BRCA2* Variants in Northeastern Japan: A Cohort Study

**DOI:** 10.1002/cam4.70443

**Published:** 2025-04-18

**Authors:** Hidekazu Shirota, Akimitsu Miyake, Maako Kawamura, Shuhei Suzuki, Kensuke Saito, Jun Yasuda, Hiroyuki Shibata, Motonobu Saito, Takeshi Iwaya, Hiroshi Tada, Muneaki Shimada, Naoki Kawamorita, Masayuki Kanamori, Eisaku Miyauchi, Hidetaka Niizuma, Tomoyuki Iwasaki, Yuki Kasahara, Hiroo Imai, Ken Saijo, Keigo Komine, Masanobu Takahashi, Tetsuya Niihori, Yoko Aoki, Toru Furukawa, Gen Tamiya, Chikashi Ishioka

**Affiliations:** ^1^ Department of Medical Oncology Tohoku University Hospital Sendai Japan; ^2^ Tohoku Medical Megabank Organization Tohoku University Sendai Japan; ^3^ Personalized Medicine Center Tohoku University Hospital Sendai Japan; ^4^ Department of Clinical Oncology Yamagata University Faculty of Medicine Yamagata Japan; ^5^ Department of Medical Oncology Hirosaki University Graduate School of Medicine Hirosaki Japan; ^6^ Division of Molecular Cellular Oncology Miyagi Cancer Center Research Institute Natori Japan; ^7^ Department of Clinical Oncology, Graduate School of Medicine Akita University Akita Japan; ^8^ Department of Gastrointestinal Tract Surgery Fukushima Medical University School of Medicine Fukushima Japan; ^9^ Department of Clinical Oncology Iwate Medical University School of Medicine Morioka Japan; ^10^ Department of Breast and Endocrine Surgical Oncology Tohoku University Graduate School of Medicine Sendai Japan; ^11^ Department of Obstetrics and Gynecology Tohoku University School of Medicine Sendai Japan; ^12^ Department of Urology Tohoku University School of Medicine Sendai Japan; ^13^ Department of Neurosurgery Tohoku University School of Medicine Sendai Japan; ^14^ Department of Respiratory Medicine Tohoku University Graduate School of Medicine Sendai Japan; ^15^ Department of Pediatrics Tohoku University School of Medicine Sendai Japan; ^16^ Department of Medical Genetics Tohoku University Graduate School of Medicine Sendai Japan; ^17^ Department of Investigative Pathology Tohoku University Graduate School of Medicine Sendai Japan

**Keywords:** BRCA1, BRCA2, CGP test, geographical distribution, solid tumor

## Abstract

**Background:**

Germline mutations in *BRCA1*/*2* are known to cause hereditary tumors in the breast, ovary, and other organs. With the widespread adoption of comprehensive diagnostics, including comprehensive genomic profiling (CGP) tests for solid tumors, many patients with *BRCA1*/*2* variants have been identified.

**Methods:**

In this study, we extracted and analyzed cases of *BRCA1*/*2* variants that were presumed to be germline, which were repeatedly detected using the CGP test for solid tumors in northeastern Japan. The frequencies of *BRCA1*/*2* variants in regional areas were compared with those of healthy individuals or nationwide cancer cohorts to investigate regional distribution.

**Results:**

Our findings revealed regional disparities in *BRCA1*/*2* pathogenic germline variants, while variants of unknown significance (VUS) showed no such differences. The regional distribution of *BRCA1* and *BRCA2* variants showed distinct patterns: pathogenic variants of *BRCA1* exhibited regional differences and were less prevalent compared to VUS, whereas *BRCA2* variants, including both pathogenic variants and VUS, did not exhibit such clear regional localization. This discrepancy in regional distribution between *BRCA1* and *BRCA2* variants could be attributed to factors such as the diversity of the genome, gender differences, and cancer types.

**Conclusions:**

These results highlight the importance of considering regional differences in comparative cohort studies, particularly in assessing the differential extension of mutations in pathogenic changes and VUS. Moreover, a presumption of pathogenicity variants would need to be discussed at the regional level.

## Graphical abstract

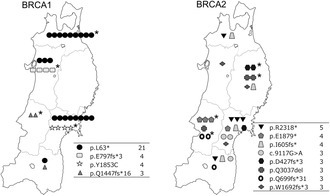

**Abbreviations**
C‐CAT: Center for Cancer Genomics and Advanced Therapeutics
CGP: comprehensive genomic profiling
F1 CDx: FoundationOne CDx
F1 liquid CDx: FoundationOne liquid CDx genome profiling
HBOC: hereditary breast and ovarian cancer syndrome
MTB: molecular tumor board
NCC Oncopanel: OncoGuide NCC Oncopanel System
VAF: variant allele frequency
VUS: variants of unknown significance

## 1 Introduction

Pathogenic germline mutations in *BRCA1* and *BRCA2* are known to cause hereditary breast and ovarian cancer syndrome (HBOC), leading to cancer development at a relatively young age [1]. Since the 1990s, these genes have been identified as inherited through familial accumulation of mutations, and *BRCA* gene testing has been conducted for patients and families seeking cancer risk evaluation [2, 3]. Although the estimated frequency of pathogenic variants of *BRCA1*/*2* is 1 in 400–500 individuals, the actual epidemiological situation remains unclear [4]. Previous large‐scale studies have shown higher risks not only for breast and ovarian cancer but also for various other cancers [5]. Recently, *BRCA1*/*2* gene testing has become available for identifying therapeutic targets, such as PARP inhibitors, for patients with breast and ovarian cancer [6]. Additionally, comprehensive genomic profiling (CGP) tests have been introduced in Japan through health insurance for cancer patients, and the results are discussed at the molecular tumor board (MTB) [7, 8]. This has led to the identification of numerous pathogenic variants in patients and families carrying *BRCA1*/*2* genes. Notably, there are distinct geographical distributions of *BRCA1*/*2* variants, unique to each country and race, with regional incidences observed in various cohorts [9, 10, 11, 12]. Here, we present the prevalence and geographical distribution of *BRCA1*/*2* variants in northeastern Japan, comparing it with that in the regional unaffected and nationwide cancer cohorts. We analyzed *BRCA1* and *BRCA2* for the prevalence of pathogenic variants and variants of unknown significance (VUS), providing insights into intrinsic genomic changes and genetic characteristics of *BRCA1* and *BRCA2*.

## 2 Material and Methods

### 2.1 Patients and CGP

A total of 3220 patients with solid tumors, undergoing chemotherapy and deemed suitable for insurance‐covered CGP tests by their physicians, agreed to provide data on their treatment outcomes conducted from September 2019 to May 2023. The commonly used CGP tests were FoundationOne CDx, FoundationOne liquid CDx genome profiling (F1 CDx and F1 liquid CDx; Chugai Pharmaceutical), and OncoGuide NCC Oncopanel System (NCC Oncopanel; Sysmex Corporation) [13, 14, 15]. F1 CDx and NCC Oncopanel are designed for tumor tissue specimens, while F1 liquid CDx assesses circulating cell‐free DNA from plasma. F1 CDx is a CGP assay developed for analyzing tumor tissues. F1 and NCC gene panel tests report genetic alterations identified in NGS analysis in variant call format (VCF) files using the company's proprietary algorithm. These variants include not only those identified as pathogenic but also changes that have not been adequately documented in scientific literature and are known as VUS. Particularly, less frequent variants (< 0.02) showing genetic polymorphism are consistently reported. Variants with some frequency from the national C‐CAT cohort were reported by all three CGP tests. Almost no differences in detection frequency were observed among tests (data not shown).

### 2.2 Regional and Nationwide Cancer Cohorts

**FIGURE 1:**
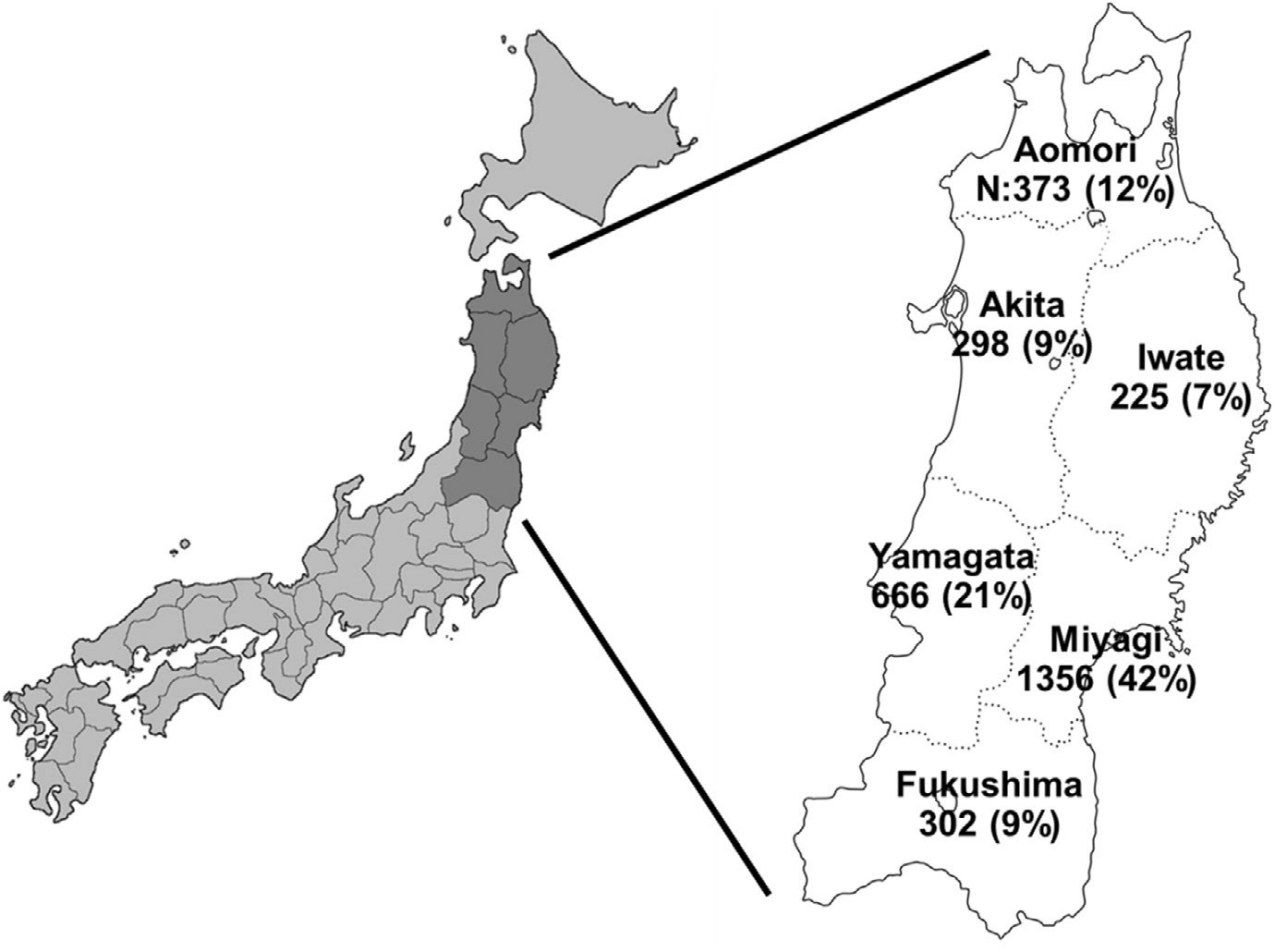
Map showing the number of comprehensive genomic profiling tests in each prefecture.

Data for CGP testing were collected and included in the analysis from three facilities: Tohoku University Hospital, Yamagata University Hospital, and Hirosaki University Hospital. These facilities are considered to represent the majority of cases of CGP testing in northeastern Japan, as MTB is provided only at these three facilities [7, 8]. The number of cases reported in each prefecture is reflected in Figure 1, while Table 1 displays the number and proportional distribution of CGP tests, gender, and types of tumors. Data (49,061 cancer patients) on the nationwide cancer cohort from the same period were extracted from the Center for Cancer Genomics and Advanced Therapeutics (C‐CAT) database [16]. The patient backgrounds (sex ratio and cancer type) are outlined in Table 1 and were comparatively analyzed with the regional and nationwide cancer cohorts. Raw data for this research were obtained from the C‐CAT Research‐Use Portal site (https://www.ncc.go.jp/en/c_cat/use/index.html). The use of this data was subject to institutional and C‐CAT data utilization reviews and was used only after receiving ethical approval.

**TABLE 1:** Characteristics of patients, CGP test, and type of cancer in regional cancer cohort.

Median (range) age	63 (0–90)
Sex	
Male (%)	1661 (51.5)
Female (%)	1559 (48.5)
CGP test	
FoundationOne CDx (%)	2467 (76.6)
FoundationOne liquid CDx (%)	559 (17.4)
NCC Oncopanel (%)	194 (4)
Total	3220

Type of cancer	Regional no. (%)	Nationwide no. (%)
Bowel	542 (16.8)	8354 (16.7)
Pancreatic	405 (12.5)	6768 (13.5)
Bile duct	265 (8.2)	4165 (8.3)
Esophagus/stomach	233 (7.2)	3073 (6.1)
Soft tissue	225 (7.0)	2200 (4.4)
Prostate	212 (6.6)	3057 (6.1)
Breast	192 (5.9)	3189 (6.4)
Ovarian	175 (5.4)	2901 (5.8)
Lung	159 (4.9)	2801 (5.6)
Uterus	156 (4.8)	1844 (3.7)
Head and neck	125 (3.9)	1781 (3.6)
Others	531 (16.4)	9842 (19.7)
Total	3220	49,975

### 2.3 Data Availability on Regional Healthy Cohort

The healthy cohort was identified from the Tohoku Medical Megabank Organization (ToMMo) project's database of 38,720 healthy individuals in Miyagi and Iwate Prefecture, also located in the middle of the northeastern (Tohoku) region and represent about 50% of the regional cancer cohort [17]. These data were retrieved from the portal site of the Japanese Multi Omics Reference Panel (jMorp, https://jmorp.megabank.tohoku.ac.jp/202001/). This database provides allele units and has been corrected to represent the number of individuals.

### 2.4 Sampling of Presumed Germline *BRCA1* /*2* Variants and Assessment of Pathogenicity

**FIGURE 2:**
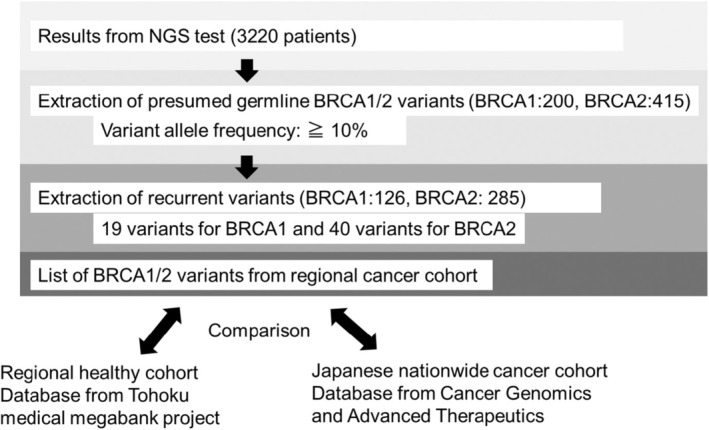
Flow diagram showing the data from *BRCA1*/*2* extraction to comparative analysis.

Figure 2 shows the process of extraction of germline *BRCA1*/*2* variants from the regional cancer cohort. NCC Oncopanel evaluation can confirm a germline *BRCA1*/*2* variant [15]. However, F1 and F1 Liquid, which are tumor‐only panels, cannot determine germline variants. Based on the Kosugi group's protocol for evaluating Presumed Germline Pathogenic Variants, high variant allele frequency (VAF) *BRCA1*/*2* variants with 10% or more were included in the analysis, categorized as suspected germline (*BRCA1*: 200 variants, *BRCA2*: 415 variants) [8, 18, 19]. Among them, cases with presumed pathogenic germline variants of BRCA1/2 which were repeatedly detected (*n* > 2), were extracted for analysis (*BRCA1*: 126 cases, *BRCA2*: 285 cases). These variants were outlined in the reports of these three CGP tests and were linked to several registries in the ToMMo healthy cohort, genome Aggregation Database (gnomAD), C‐CAT data, and *BRCA* Exchange [16, 17, 20, 21]. All patients with pathogenic germline variants of *BRCA1*/*2* also reviewed detailed family histories but did not include the same family members. This is based on interviews with the attending physician and genetic counselor. Up to the second‐degree of relative is verified. All CGP tests were discussed by MTB, and the pathogenic significance of *BRCA1*/*2* was determined [8]. For this discussion, public databases such as ClinVar were referenced to define whether germline pathogenic variants were presumed for *BRCA1*/*2*.

### 2.5 Statistical Methods

To compare the proportions of variant observations across cohorts, Fisher's exact test was utilized, with a two‐tailed test significance level set at *p* < 0.05. Additionally, the Clopper–Pearson method was used to calculate exact 95% confidence intervals for these proportions. The expected number of variants in each prefecture of the Northeast region (Tohoku) was estimated using variant frequencies from nationwide cancer cohorts. A Poisson conditional autoregressive (CAR) model, accounting for spatial autocorrelation between prefectures, was employed to derive 95% Bayesian prediction intervals from these expected counts. This approach assessed the clustering of variants within each prefecture. Our analysis focused on variant types observed three or more times in any Northeast region prefecture, conducted separately for each type. All statistical analyses were conducted using R 4.3.1.

### 2.6 Ethics Approval

This study was approved by the Institutional Review Board (IRB) of the Tohoku University Hospital (IRB No. 2021‐1‐250 250 and 2023‐1‐1036) and was performed in compliance with the Helsinki Declaration of 1964 and later versions. Informed consent to use the results for research was obtained from all patients, but more detailed study protocols were approved by the Institutional Reviewer Board (IRB) through a waiver of informed consent.

## 3 Results

### 3.1 Sampling of Presumed Germline *BRCA1* /*2* Variants From Regional Cancer Cohort

The northeast region of Japan (Tohoku), the focus site of this study, comprised 20% of the total area of Japan and 8% of the entire population (Figure 1). The region is predominantly mountainous, with mostly rural countryside and modest population inflow, coupled with a significant issue of population diminishment. Population mobility in other regions is presumed to be low compared to urban areas. Large‐scale SNP analysis has revealed unique genetic features in northeastern Japan [22].

CGP tests for clinical purposes were conducted in six prefectures of the northeast region of Japan. These tests excluded the assessment of major polymorphisms of *BRCA1*/*2* genes using a proprietary algorithm. Therefore, the detected *BRCA1*/*2* variants are predominantly somatic and/or minor germline mutations, including pathogenic variants and VUS. We extracted presumed germline variants from them, as described in the Materials and Methods section. Moreover, potential germline *BRCA1*/*2* variants with high VAF and a frequency of 2 or higher were extracted from 3220 CGP tests performed in the northeast region, constituting the regional cancer cohort (Figure 2). Nineteen variants for *BRCA1* and 40 variants for *BRCA2* were covered by the analysis, including six pathogenic variants for *BRCA1* and 12 for *BRCA2*. Notably, *BRCA1* had fewer variants in both the pathogenic and VUS groups compared to *BRCA2*. Regarding cancer type and gender in pathogenic variants, *BRCA1* was more commonly associated with breast and ovarian cancer, while *BRCA2* was more widely detected in prostate cancer, pancreatic cancer, cholangiocarcinoma, and others (Table 2). *BRCA1* was more frequently observed in women than in men, while *BRCA2* had a nearly equal gender ratio. These findings are consistent with previous reports [5, 23]. As there were differences in the number of variants in *BRCA1* and *BRCA2*, gender ratios, and cancer types, we compared these outcomes separately.

**TABLE 2:** List of pathogenic variants of BRCA1/2 and the number of cancer types.

	*N*	Type of cancer (no.)
BRCA1 variant		
p.L63*	21	Breast 4, ovarian 10, skin 2, endometrial 2, gastric1, colorectal 1, CUP1
p.E797fs*3	4	Breast 3, cervical 1
p.Y1853C	4	Ovarian 3, breast 1
p.Q1447fs*16	3	Ovarian 3
p.A1789T	2	Ovarian 1, colorectal 1
p.S426fs*10	2	Pancreatic 2
Total	36	Breast 8, ovarian 17, pancreatic 2, others 7
Male (%)	4 (11.1)	
Female (%)	32 (88.9)	
BRCA2 variant		
p.R2318*	5	Ovarian 1, prostate 1, lung 1, sarcoma 1, pancreatic 1
p.E1879*	4	Breast 2, ovarian 1, cervical 1
p.I605fs*9	4	Small intestine 1, sarcoma 1, esophagus 1, bile duct 1
p.D427fs*3	3	Prostate 2, pancreatic 1
c.9117G>A	3	Prostate 1, bile duct 1, endometrium 1
p.Q3037del	3	Prostate 2, ovarian 1
p.Q699fs*31	3	Prostate 2, breast 1
p.W1692fs*3	3	Prostate 2, esophagus 1
p.I1859fs*3	2	Breast 2
p.E1550fs*4	2	Pancreatic 1, colorectal 1
p.K1191fs*6	2	Pancreatic 2
p.T3033fs*29	2	Breast 1, pancreatic 1
Total	36	Breast 5, ovarian 3, prostate 10, pancreatic 6. bile duct 2, others 9
Male (%)	19 (52.8)	
Female (%)	17 (47.2)	

### 3.2 Differences in the Frequency of Each Variant of *BRCA1* /*2* Between the Cancer and Healthy Cohorts

Table 3 presents the number and percentage of *BRCA1* variants identified in 3220 cases of solid tumors at the local level. Additionally, minor variants detected in three or more cases are included. Variants detected in two cases are shown in a Table S1, since the data tends to become more blurred as the number of cases decreases. In a separate column, the number and percentage of these variants in a healthy cohort, comprising approximately 38,000 individuals residing in an area that geographically includes a part of the regional cancer cohort are displayed, along with the ratios between the cohorts in Table 3. We observed a significant enrichment of pathogenic variants (approximately 5‐fold higher) in the cancer cohort compared to the healthy cohort. In contrast, the ratios of VUS variants, considered benign, were compatible (approximately 1.0) with the levels observed in the healthy cohort.

**TABLE 3:** Number and frequency of three or more detected *BRCA1* variants in three cohorts.

BRCA1		Regional cancer cohort (Tohoku)		Regional healthy cohort (ToMMo)		Cancer cohort: healthy cohort		Nationwide cancer cohort without Tohoku		Regional: nationwide	
		Total 3220		Total 38,720				Total 49,061			
Variant	Annotation	*n*	Rate	*n*	Rate	Ratio	*p*	*n*	Rate	Ratio	*p*
p.S1577P	VUS	31	0.963	398	1.028	0.94		450	0.917	1.050	
p.L52F	VUS	26	0.807	254	0.656	1.23		299	0.609	1.325	
p.L63*	Pathogenic	21	0.652	34	0.088	7.43	***	66	0.135	4.848	***
p.P209L	VUS	10	0.311	93	0.240	1.29		58	0.118	2.627	**
p.E797fs*3	Pathogenic	4	0.124	3	0.008	16.03	**	9	0.018	6.772	***
p.Y1853C	Pathogenic	4	0.124	8	0.021	6.01	*	11	0.022	5.540	**
p.Q1447fs*16	Pathogenic	3	0.093	1	0.003	36.07	*	2	0.004	22.855	***
p.F1662S	VUS	3	0.093	27	0.070	1.34		62	0.126	0.737	
p.N909I	VUS	3	0.093	27	0.070	1.34		37	0.075	1.235	
p.T843A	VUS	3	0.093	14	0.036	2.58		17	0.035	2.689	

*Note:* *p < 0.05, **p < 0.01, ***p < 0.001.

Table 4 illustrates the number and distribution of *BRCA2* variants, which exhibit more variability than those of *BRCA1* but demonstrate similar tendencies. Moreover, pathogenic variants were dozens of times more common in the cancer cohort, while VUS were present at approximately the same frequency as in the healthy cohort. The less frequent variants had larger ratio deviations.

**TABLE 4:** Number and frequency of three or more detected *BRCA2* variants in three cohorts.

BRCA2		Regional cancer cohort (Tohoku)		Regional healthy cohort (ToMMo)		Cancer cohort: healthy cohort		Nationwide cancer cohort without Tohoku		Regional: nationwide	
		Total 3220		Total 38,720				Total 49,061			
Variant	Annotation	*n*	Rate	*n*	Rate	Ratio	*p*	*n*	Rate	Ratio	*p*
p.K322Q	VUS	71	2.205	915	2.363	0.93		1016	2.071	1.06	
p.V2109I	VUS	50	1.553	522	1.348	1.15		616	1.256	1.24	
p.T582P	VUS	21	0.652	213	0.550	1.19		307	0.626	1.04	
p.K1132R	VUS	17	0.528	134	0.346	1.53		103	0.210	2.51	**
p.N2436I	VUS	14	0.435	294	0.759	0.57	*	241	0.491	0.89	
p.G3210S	VUS	9	0.280	96	0.248	1.13		9	0.018	15.24	***
p.A2351G	VUS	6	0.186	77	0.199	0.94		123	0.251	0.74	
p.I770V	VUS	6	0.186	56	0.145	1.29		26	0.053	3.52	*
p.N1330S	VUS	6	0.186	23	0.059	3.14	*	7	0.014	13.06	***
p.R2318*	Pathogenic	5	0.155	25	0.065	2.40		124	0.253	0.61	
p.V208G	VUS	5	0.155	21	0.054	2.86	*	41	0.084	1.86	
p.E1879*	Pathogenic	4	0.124	1	0.003	48.10	***	4	0.008	15.24	***
p.I605fs*9	Pathogenic	4	0.124	5	0.013	9.62	**	35	0.071	1.74	
c.9117G>A	Pathogenic	3	0.093	2	0.005	18.04	***	9	0.018	5.08	*
p.D427fs*3	Pathogenic	3	0.093	1	0.003	36.07	***	12	0.024	3.81	
p.Q3037del	Pathogenic	3	0.093	2	0.005	18.04	**	3	0.006	15.24	**
p.W1692fs*3	Pathogenic	3	0.093	1	0.003	36.07	**	17	0.035	2.69	
p.Q699fs*31	Pathogenic	3	0.093	0	NA	NA		1	0.002	48.71	**
p.E3377D	VUS	3	0.093	27	0.070	1.34		34	0.069	1.34	
p.V1810I	VUS	3	0.093	2	0.005	18.04	**	1	0.002	45.71	***

*Note:* *p < 0.05, **p < 0.01, ***p < 0.001.

### 3.3 Differences in the Frequency of Each Variant of *BRCA1* /*2* Between Regional Cancer and Nationwide Cancer Cohorts

The frequency of these variants was determined from the C‐CAT database for nationwide CGP test results in patients with solid tumors across the country. A comparative geographic analysis between northeastern Japan and the nationwide cohort was then conducted. The percentages of solid cancer types in the regional and nationwide cancer cohorts were comparable, as shown in Table 1, suggesting that the difference in variance among cancer types evaluated between the two cohorts is negligible.

The separate column shows the number and frequency of each variant in the nationwide cancer cohort, excluding those from the northeast region, along with the ratio between the regional and nationwide cohorts. All of the *BRCA1* pathogenic variants had approximately five times higher frequency in the regional cohort compared to the nationwide cohort (Table 3). Conversely, VUS had almost comparable values between the two cohorts. Statistically higher values were detected for all pathogenic variants except for p.P209L in the VUS.

Pathogenic *BRCA1* variants were disproportionately distributed in northeastern Japan, while the detection of VUS occurred at a comparable rate nationwide. However, for *BRCA2*, no such tendency was observed as a whole (Table 4). In contrast, a varied pattern was observed in *BRCA2*, with four out of the eight pathogenic variants being significantly more frequently detected in the regional cohort compared to the nationwide cancer cohort, and a similar tendency was observed for five of the 12 VUS variants. In comparison with the nationwide cancer cohort for *BRCA2*, three of the seven pathogenic variants were more commonly found in the regional area, and for VUS, five of the 12 variants were significantly more frequent. The p.R2318* variant had a ratio of less than 1, suggesting that it was a rare type in regional areas. This variant was the only one that did not differ significantly from healthy controls.

**FIGURE 3:**
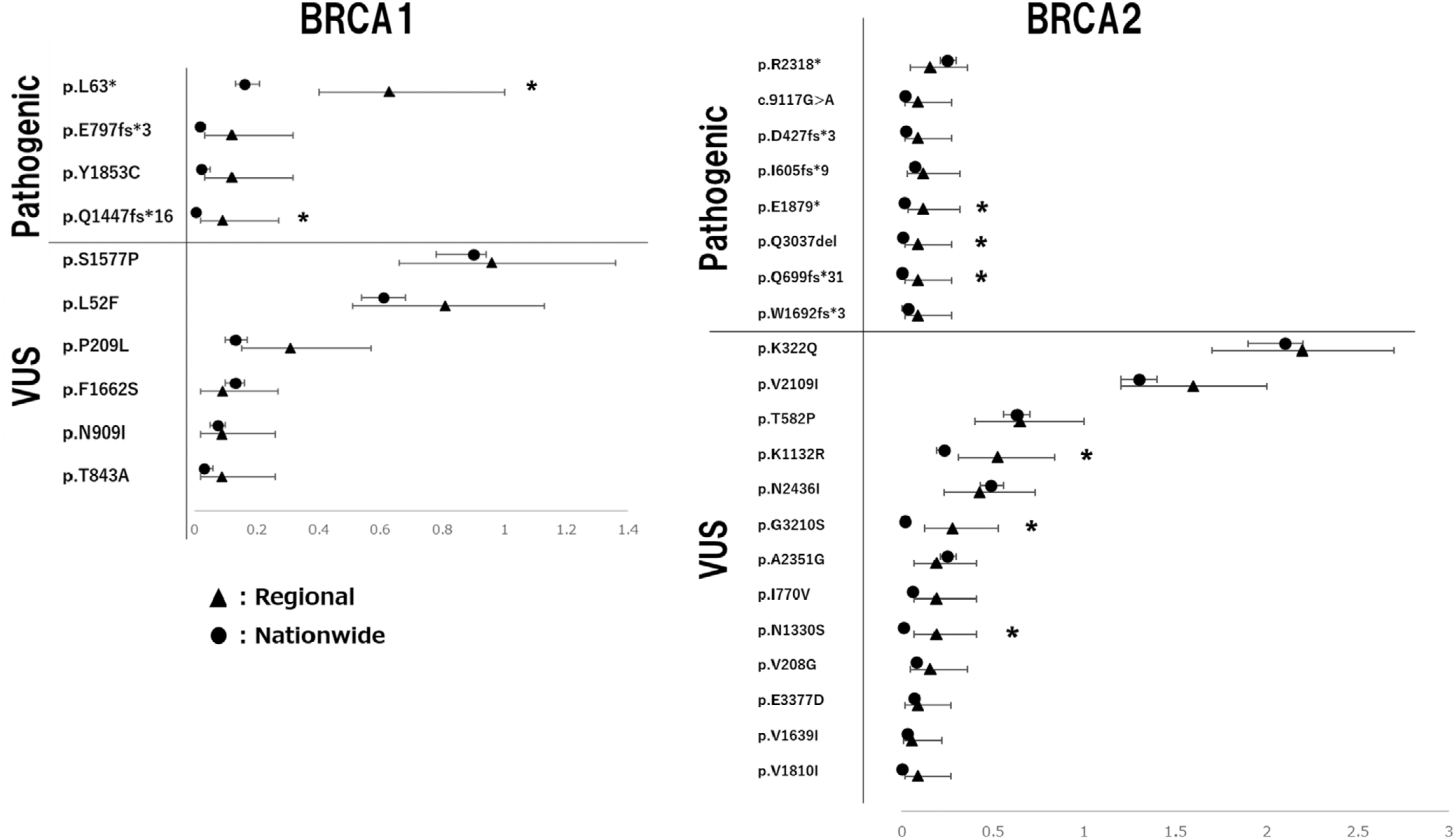
Confidence intervals from observed counts of BRCA1/2 variants between the two groups. An exact confidence interval was obtained to visualize the possible differences between regional and nationwide cancer cohorts. Variants without overlapping confidence intervals between the two groups were marked with *.

Another statistical analysis, using the Clopper–Pearson method, was used to compare the frequency rates between the regional and nationwide cancer cohorts. An exact confidence interval was obtained to visualize the possible differences between the two groups (Figure 3). This method is suitable for comparing data that do not follow a normal distribution and have a low percentage of observations. As shown in Figure 3, for pathogenic variants in *BRCA1* and *BRCA2*, the differences between the two groups showed almost no overlapping confidence intervals, except for p.R2318*. Conversely, for VUS, many variants had overlapping confidence intervals.

### 3.4 Differences in the Frequency of Each *BRCA1* /*2* Variant by Prefecture and Their Clustering

**FIGURE 4:**
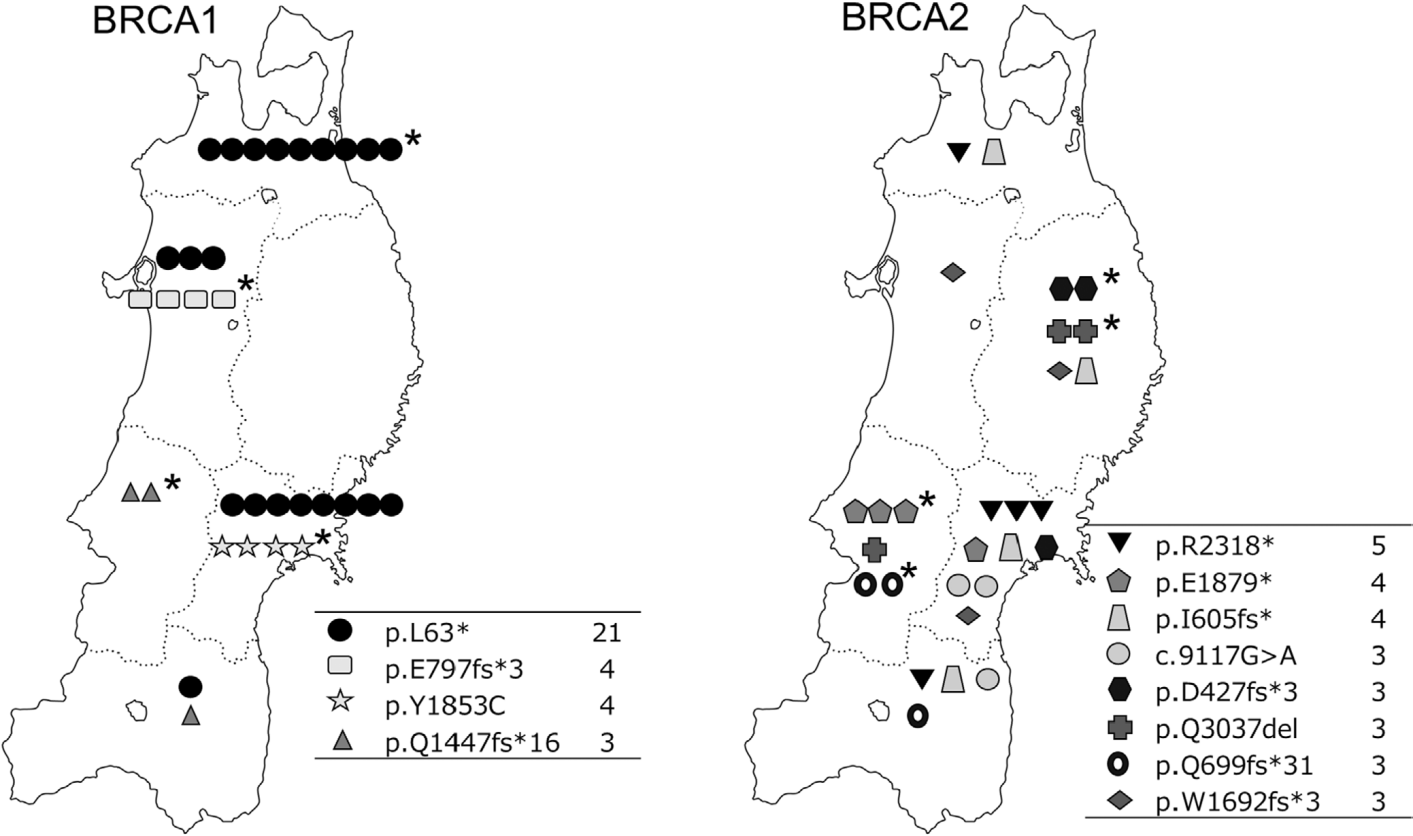
Geographical distribution of the number of pathogenic variants of *BRCA1*/*2* by prefecture. Plots with detections above the predicted values (95% Bayesian prediction interval) were marked with *.

The results indicated a higher incidence of pathogenic variants of *BRCA1*/*2* at a regional level. Next, we explored whether there was a regional aggregation of these variants in each prefecture. A method was applied to determine the expected number of cases (95% Bayesian prediction interval) according to each variant and prefecture. One example is the formula for the *BRCA1* L63* variant reflected in Figure S1. If the actual number of observations exceeded the upper limit, the presence of regional agglomeration was considered. The number of variant cases was plotted for each prefecture on a map displayed in Figure 4. Plots with detections above the predicted values were marked with a star (the calculations for the individual variants are shown in the Figure S2). All four variants for *BRCA1* were found to be concentrated in one prefecture, as were four of the eight for *BRCA2*. This indicates that the incidence is not equally distributed among the prefectures but instead is regional and concentrated.

## 4 Discussion

This study compared the frequency of presumed germline *BRCA1*/*2* variants identified by CGP testing in the regional area of Japan with that in the regional healthy and/or nationwide cancer cohorts. This comparison included non‐pathogenic VUS. Pathogenic variants of *BRCA1*/*2* were highly abundant in cancer cohorts compared to the healthy cohort. In contrast, VUS were observed at almost the same frequency as in the healthy cohort (Tables 3 and 4). A comparison of regional and nationwide cancer cohorts revealed that *BRCA1*/*2* pathogenic variants tended to exceed the expected number between the cohorts, indicating a regional difference (Tables 3 and 4, Figure 3). However, for *BRCA1*/*2* VUS, there was mostly no clear difference in the expected number of variants between cohorts. Furthermore, pathogenic *BRCA1*/*2* variants were concentrated in a single prefecture (Figure 4). By including not only pathogenic variants but also VUS in this analysis, we could compare the spreading patterns of the former and investigate the characteristics of *BRCA1*/*2* genes.

The study involved comparisons with a healthy cohort. The frequency of pathogenic variants was significantly higher in the cancer cohort, while VUSs were at a comparable level (with a ratio around 1.0; Tables 3 and 4). These results were expected when comparing healthy and cancer cohorts, and conversely, this difference provided an estimate of pathogenic significance. While VUS showed little difference compared to the nationwide cancer cohort, there was a large variation in frequency for pathogenic variants. This finding highlighted the importance of cross‐comparing regional characteristics in genetic variations for diseases, such as cancers in cohort‐to‐cohort studies, even if there are no significant changes in genetic polymorphisms or other changes of non‐pathogenic origin.

Previous studies conducted in various countries have indicated that pathogenic variants of *BRCA1*/*2* exhibit variability in incidence among different regions [9, 10, 11, 12]. In this study, we observed a distinct local regional pattern in the distribution of pathogenic variants within the Japanese region. In contrast, several *BRCA1*/*2* variants that are frequent nationwide, as reported in C‐CAT data, were not detected in more than two cases in our study region. These variants included *BRCA1* Q934* (*n* = 44), *BRCA1* K654fs*47 (*n* = 15), *BRCA1* E1214* (*n* = 14), *BRCA2* I1859fs*3 (*n* = 122), *BRCA2* S1882* (*n* = 35), *BRCA2* Q3026* (*n* = 32), *BRCA2* S2835* (*n* = 26), *BRCA2* N2135fs*3 (*n* = 21), *BRCA2* L1908fs*2 (*n* = 13), *BRCA2* A2185fs*2 (*n* = 13), *BRCA2* T3033fs*2 (*n* = 13), and *BRCA2* R2520* (*n* = 10). These findings suggest that specific regional variations exist not only in the northeast region but also across the entire country, indicating that other germline pathogenic variants are also likely to exhibit regional variations. Currently, MTBs are held in each region to discuss the results of CGP tests as part of the Japanese health insurance system [7, 8]. There is ongoing consideration regarding the standardization and centralization of information in a location. However, pathogenic variants in the germline, such as *BRCA1*/*2*, are characterized by regional specificity. Sometimes it is difficult to evaluate the pathogenic significance of minor variants. The discussions in MTB are based on the patient's medical history, family history, and genome‐wide instability, not just the genetic sequence. Thus, case experience in MTB is important. Variants that are repeatedly observed only in a limited geographic area are easy to determine pathogenicity. Therefore, we believe it is reasonable to discuss them at the regional level to better understand their implications and facilitate appropriate management strategies.

It is important to distinguish between the spread of pathogenic genes and VUS within a specific region. This study did not identify significant rare VUS locally. When comparing the three cohorts of this study, few differences were observed (Tables 3 and 4). This regional tendency suggests the presence of non‐spread (localized) or relatively new variants. Patients with pathogenic variants typically have an onset of cancer at a young age, and most have a significant family and medical history of cancer, which can sometimes complicate the observation of family lineage continuity [1, 4, 5]. In contrast, VUS would be expected to gradually distribute across the country without barriers over many generations. While pathogenic variants may extend over time, their spread is likely to occur at a slower pace and may even face the possibility of elimination.

Both *BRCA1* and *BRCA2* alterations serve as a pathogenic basis for HBOC and are associated with increased susceptibility to breast or ovarian cancer [1]. Functionally, they are involved in DNA damage repair through homologous recombination, and in clinical settings, PARP inhibitors have shown effectiveness, indicating their similarity in function. However, there is little homology between the two genes, and many functional differences have been reported [24]. Notably, the number of variants in *BRCA2* is significantly higher than in *BRCA1*, which may indicate that the genome is more diverse in the *BRCA2* gene. In our comparison of regional differences, all detected *BRCA1* pathogenic variants were clustered in the northeast region of Japan, exhibiting a distinct geographic pattern by prefecture. In contrast, *BRCA2* variants appeared to vary regardless of the specific pathogenic variants (Tables 3 and 4 and Figures 3 and 4). Some pathogenic variants of *BRCA2* did not show significant regional differences, while certain types of VUS of *BRCA2* exhibited regional disparities, suggesting that these variants may be relatively new generations and have not yet spread to the rest of the country. In contrast, the *BRCA1* variants in this cohort are considered to be older‐generation changes that have been inherited over many generations. Therefore, men with pathogenic *BRCA1* variants are not characterized by a high incidence of cancer and therefore have a higher chance of survival (Table 2). Conversely, pathogenic variants in *BRCA2* lack stability and are more likely to appear, but they may also be more prone to elimination due to a comparable cancer incidence between males and females.

There are some limitations in our analysis. First, most CGP tests used in this study are tumor‐only panels, meaning they cannot confirm germline variants. Therefore, there is a possibility that somatic mutations may have been included in our analysis. The VAF of most liquid samples are concentrated at 0.5, and those of tissue samples considerably vary, making it difficult to filter by VAF alone (data not shown). While we have checked all pathogenic variants, most patients had medical and family histories of cancer, leading us to suspect germline mutations during our MTB discussions [7, 8]. However, the number and percentage of pathogenic variants in this study may not vary significantly due to these limitations. Additionally, the frequency of VUS in our study was almost identical to that of the normal healthy cohort in the same region. While we could rule out the possibility of somatic mutation contamination, the statistical range of these findings was considered almost negligible. Moreover, another limitation is related to the assumption that the same variant in this study had originated and expanded from the same ancestor. While past reports have established the existence of founder mutations for a substantial number of *BRCA1*/*2* variants, individual occurrences of the same variants, including VUS may exist in various parts of the country [9, 10, 11, 12, 25]. However, the gnomAD, a comprehensive genomic database, shows that these variants, including VUS, are rarely detected outside of East Asia. Therefore, these variants may have originated in Japan from the same ancestor. In addition, the cancer cohort in this study does not reflect all cancer patients. The cohort was selected for the poor prognosis group of metastatic and recurrent cases. In other words, few cancers in the good prognosis group are included in this cohort. Furthermore, the issue of the sample size is a limitation of our study. We analyzed a regional cohort of 3220 cancer patients which was compared with a healthy cohort of 40,000 residents in the same region and a nationwide cohort of 50,000 cancer patients. The limited number of variants in each cohort, particularly for rare variants (*n* = 2 or 3), contributed to the large variance of the data. In future studies, including more cases and conducting deeper comparisons with other regional cohorts, may allow for a more comprehensive analysis of the prevalence and selection of pathogenic variants of *BRCA1*/*2*, as well as genetic differences.

## Author Contributions

**Hidekazu Shirota:** conceptualization (equal), data curation (equal), formal analysis (equal), investigation (equal), methodology (equal), project administration (equal), resources (equal), visualization (equal), writing – original draft (equal). **Akimitsu Miyake:** data curation (equal), formal analysis (equal). **Maako Kawamura:** data curation (equal), formal analysis (equal). **Shuhei Suzuki:** project administration (equal), resources (equal). **Kensuke Saito:** project administration (equal). **Jun Yasuda:** investigation (equal), project administration (equal), resources (equal). **Hiroyuki Shibata:** project administration (equal), resources (equal). **Motonobu Saito:** project administration (equal), resources (equal). **Takeshi Iwaya:** project administration (equal), resources (equal). **Hiroshi Tada:** project administration (equal). **Muneaki Shimada:** project administration (equal). **Naoki Kawamorita:** project administration (equal). **Masayuki Kanamori:** project administration (equal). **Eisaku Miyauchi:** project administration (equal). **Hidetaka Niizuma:** project administration (equal). **Tomoyuki Iwasaki:** project administration (equal). **Yuki Kasahara:** project administration (equal). **Hiroo Imai:** project administration (equal). **Ken Saijo:** project administration (equal). **Keigo Komine:** project administration (equal). **Masanobu Takahashi:** project administration (equal). **Tetsuya Niihori:** project administration (equal). **Yoko Aoki:** project administration (equal). **Toru Furukawa:** conceptualization (equal). **Gen Tamiya:** conceptualization (equal), investigation (equal), project administration (equal), writing – original draft (equal). **Chikashi Ishioka:** project administration (equal).

## Disclosure

This research received no external funding.

## Ethics Statement

Approval of the research protocol and ethics by an Institutional Reviewer Board: IRB No. 2021‐1‐250 and 2023‐1‐1036. All patients obtained Informed consent for research, but more detailed study protocols were approved by the IRB through the disclosure of information.

## Conflicts of Interest

Dr. Ishioka has received scholarship (incentive) endowments from Takeda, Daiichi‐Sankyo, Ono, Asahi‐Kasei Pharma, Taiho, and Chugai, as well as a research grant from Hitachi and Riken Genesis. Dr. Takahashi has received research funding from Ono Pharmaceutical, Chugai Pharmaceutical, and MSD, lecturer fees from Daiichi Sankyo, Ono Pharmaceutical, Brystol Meyers Squibb, Taiho Pharmaceutical Company. All remaining authors declare no conflicts of interest.

## Supporting information


Figure S1.

Figure S2.

Table S1.

Data S1.

## Data Availability

Data sharing is not applicable to this article as no new data were created or analyzed in this study.
