# Supplementary material for: Regional Differences in the Frequency of BRCA1 and BRCA2 Variants in Northeastern Japan: A Cohort Study
Source: Cancer Med. 2025 Apr 18;14(8):e70443. doi: 10.1002/cam4.70443 (PMC12007429; doi:10.1002/cam4.70443)
Supplement: Supplementary file 1 — Figure S1. [file CAM4-14-e70443-s003.pptx]

## Slide 1
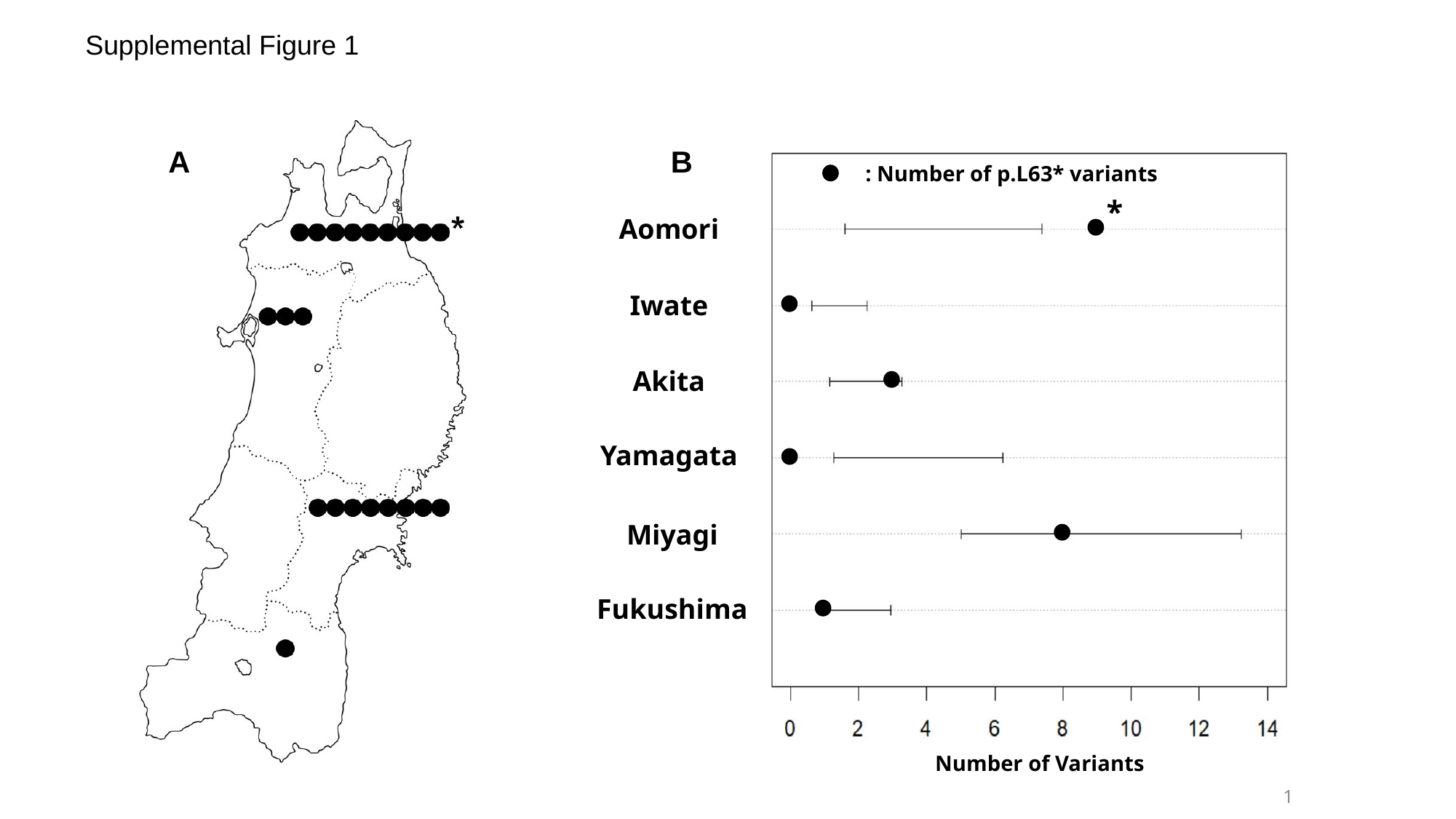

Supplemental Figure 1
A
B
: Number of p.L63* variants
*
Aomori
Iwate
Akita
Yamagata
Miyagi
Fukushima
Number of Variants
1
