# Supplementary figures and images for: Regional Differences in the Frequency of BRCA1 and BRCA2 Variants in Northeastern Japan: A Cohort Study
Source: Cancer Med. 2025 Apr 18;14(8):e70443. doi: 10.1002/cam4.70443 (PMC12007429; doi:10.1002/cam4.70443)

## Slide 1
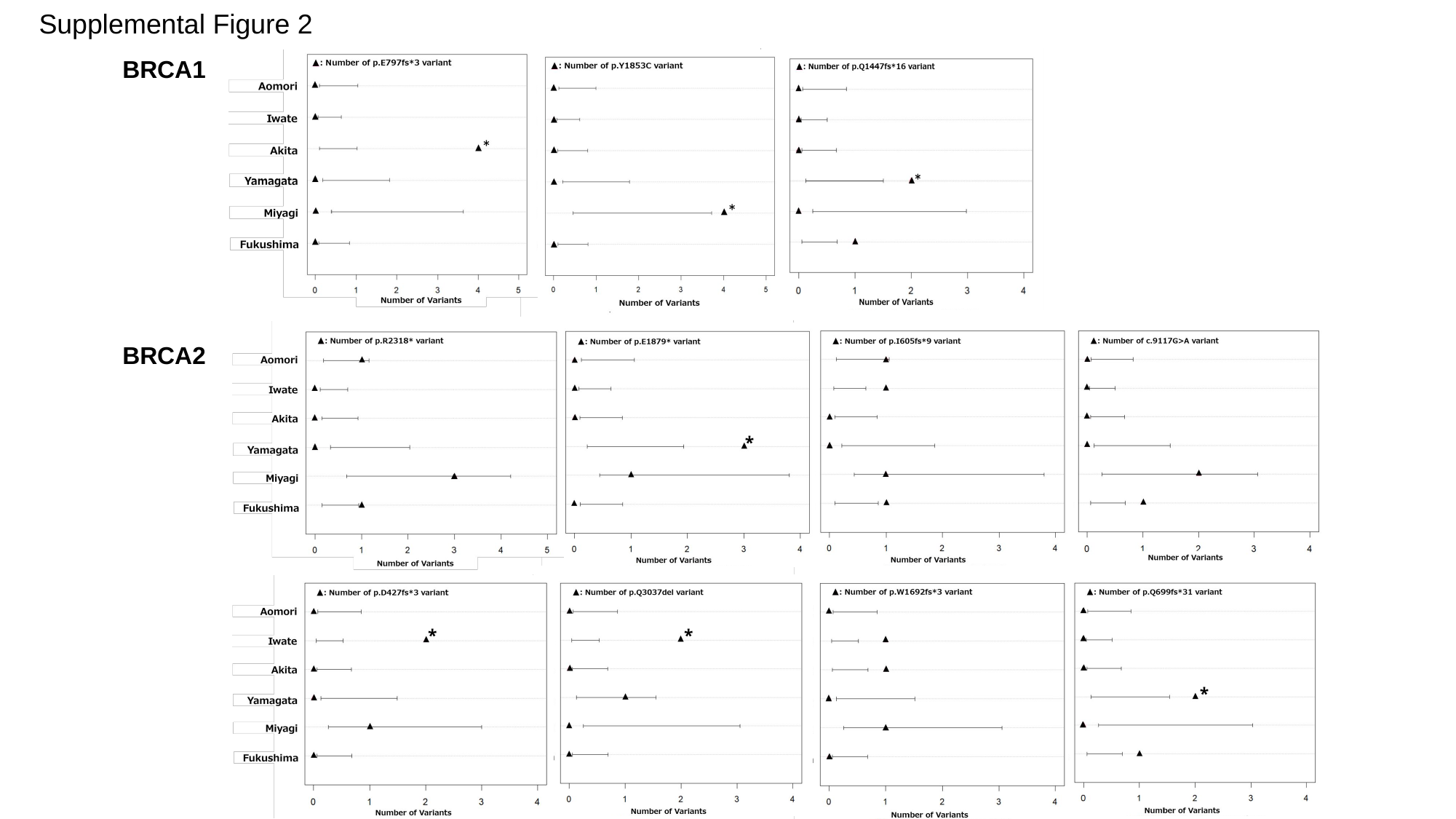

Supplemental Figure 2
BRCA1
BRCA2
*
*
*
*

Supplement: Supplementary file 2 — Figure S2. [file CAM4-14-e70443-s004.pptx]
