# Supplementary material for: Regional Differences in the Frequency of BRCA1 and BRCA2 Variants in Northeastern Japan: A Cohort Study
Source: Cancer Med. 2025 Apr 18;14(8):e70443. doi: 10.1002/cam4.70443 (PMC12007429; doi:10.1002/cam4.70443)
Supplement: Supplementary file 3 — Table S1. [file CAM4-14-e70443-s002.docx]

Supplemental Table 1. Number and frequency of two detected *BRCA1* and *BRCA2* variants in three cohorts

| *BRCA1* |  | Regional cancer cohort (Tohoku) | | Regional healthy cohort (ToMMo) | | cancer cohort : healthy cohort | | Nationwide cancer cohort without Tohoku | | Regional : Nationwide | |
| --- | --- | --- | --- | --- | --- | --- | --- | --- | --- | --- | --- |
|  |  | Total 3220 | | Total 38720 | |  |  | Total 49,061 | |  |  |
| variant | Annotation | n | Rate | n | Rate | Ratio | p. | n | Rate | Ratio | p. |
| p.A1789T | Pathogenic | 2 | 0.062 | 2 | 0.005 | 12.02 | * | 1 | 0.002 | 30.473 | ** |
| p.S426fs*10 | Pathogenic | 2 | 0.062 | 0 | NA | NA |  | 1 | 0.002 | 30.473 | ** |
| p.A1752G | VUS | 2 | 0.062 | 72 | 0.186 | 0.33 |  | 12 | 0.024 | 2.539 |  |
| p.E404D | VUS | 2 | 0.062 | 0 | NA | NA |  | 1 | 0.002 | 30.473 | ** |
| p.K1690Q | VUS | 2 | 0.062 | 11 | 0.028 | 2.19 |  | 26 | 0.053 | 1.172 |  |
| p.Q1459R | VUS | 2 | 0.062 | 0 | NA | NA |  | 17 | 0.035 | 1.793 |  |
| p.R1589H | VUS | 2 | 0.062 | 3 | 0.008 | 8.02 | * | 1 | 0.002 | 30.473 | ** |
| p.T231M | VUS | 2 | 0.062 | 1 | 0.003 | 24.05 |  | 3 | 0.006 | 10.158 | * |

| *BRCA2* |  | Regional cancer cohort (Tohoku) | | Regional healthy cohort (ToMMo) | | cancer cohort : healthy cohort | | Nationwide cancer cohort without Tohoku | | Regional : Nationwide | |
| --- | --- | --- | --- | --- | --- | --- | --- | --- | --- | --- | --- |
|  |  | Total 3220 | | Total 38720 | |  |  | Total 49,061 | |  |  |
| variant | Annotation | n | Rate | n | Rate | Ratio | p. | n | Rate | Ratio | p. |
| p.V1639I | VUS | 2 | 0.062 | 9 | 0.018 | 3.39 |  | 11 | 0.022 | 2.77 |  |
| p.I1859fs*3 | Pathogenic | 2 | 0.062 | 108 | 0.220 | 0.28 | * | 110 | 0.224 | 0.28 | * |
| p.D1618E | VUS | 2 | 0.062 | 35 | 0.071 | 0.87 |  | 37 | 0.075 | 0.82 |  |
| p.D1728N | VUS | 2 | 0.062 | 35 | 0.071 | 0.87 |  | 37 | 0.075 | 0.82 |  |
| p.E1550fs*4 | Pathogenic | 2 | 0.062 | 0 | NA | NA |  | 0 | NA | NA |  |
| p.E2275K | VUS | 2 | 0.062 | 1 | 0.002 | 30.47 | * | 3 | 0.006 | 10.16 | * |
| p.F2058L | VUS | 2 | 0.062 | 1 | 0.002 | 30.47 | * | 3 | 0.006 | 10.16 | * |
| p.G2508S | VUS | 2 | 0.062 | 43 | 0.088 | 0.71 |  | 45 | 0.092 | 0.68 |  |
| p.H523R | VUS | 2 | 0.062 | 23 | 0.047 | 1.32 |  | 25 | 0.051 | 1.22 |  |
| p.K1191fs*6 | Pathogenic | 2 | 0.062 | 0 | NA | NA |  | 0 | NA | NA |  |
| p.L184P | VUS | 2 | 0.062 | 31 | 0.063 | 0.98 |  | 33 | 0.067 | 0.92 |  |
| p.L760F | VUS | 2 | 0.062 | 23 | 0.047 | 1.32 |  | 25 | 0.051 | 1.22 |  |
| p.R1512H | VUS | 2 | 0.062 | 0 | NA | NA |  | 2 | 0.004 | 15.24 | * |
| p.S1140R | VUS | 2 | 0.062 | 10 | 0.020 | 3.05 |  | 12 | 0.024 | 2.54 |  |
| p.S2213P | VUS | 2 | 0.062 | 10 | 0.020 | 3.05 |  | 12 | 0.024 | 2.54 |  |
| p.T1887M | VUS | 2 | 0.062 | 9 | 0.018 | 3.39 |  | 11 | 0.022 | 2.77 |  |
| p.T3033fs*29 | Pathogenic | 2 | 0.062 | 9 | 0.018 | 3.39 |  | 11 | 0.022 | 2.77 |  |
| p.V2050I | VUS | 2 | 0.062 | 7 | 0.014 | 4.35 |  | 9 | 0.018 | 3.39 |  |
| p.V2503I | VUS | 2 | 0.062 | 9 | 0.0183 | 3.39 |  | 11 | 0.022 | 2.77 |  |
| p.V3081I | VUS | 2 | 0.062 | 3 | 0.0061 | 10.16 |  | 5 | 0.010 | 6.09 |  |
