# Supplementary material for: Regional Differences in the Frequency of BRCA1 and BRCA2 Variants in Northeastern Japan: A Cohort Study
Source: Cancer Med. 2025 Apr 18;14(8):e70443. doi: 10.1002/cam4.70443 (PMC12007429; doi:10.1002/cam4.70443)
Supplement: Supplementary file 4 — Data S1. [file CAM4-14-e70443-s001.docx]

Supplemental Figure 1. Predicted value and observed number of *BRCA1* p.L63* variant in each prefecture.

(A) Geographical distribution of the number of BRCA1 p.L63* variant by prefecture. (B) 95% Bayesian prediction intervals and observed number of *BRCA1* p.L63* variant in each prefecture. *: Prefecture with detections above the predicted values.

Supplemental Figure 2. Predicted value and observed number of *BRCA1/2* variants in each prefecture. *: Prefecture with detections above the predicted values.

Supplemental Table 1. Number and frequency of two detected *BRCA1* and *BRCA2* variants in three cohorts

Shaded columns are pathogenic variants. Tohoku, cancer cohort in northeast region of Japan; ToMMo, healthy cohort in northeast region of Japan. *p < 0.05, **p < 0.01.
